# Spinal pain in Danish school children – how often and how long? The CHAMPS Study-DK

**DOI:** 10.1186/s12891-017-1424-5

**Published:** 2017-03-27

**Authors:** Kristina Boe Dissing, Lise Hestbæk, Jan Hartvigsen, Christopher Williams, Steven Kamper, Eleanor Boyle, Niels Wedderkopp

**Affiliations:** 1 Department of Sports Science and Clinical Biomechanics, Faculty of Health Sciences, University of Southern Denmark, Campusvej 55, DK-5230 Odense M, Denmark; 2 Nordic Institute of Chiropractic and Clinical Biomechanics, Campusvej 55, DK-5230 Odense M, Denmark; 3 Hunter Medical Research Institute, School of Medicine and Public Health, University of Newcastle, Callaghan, NSW Australia; 4 Hunter New England Population Health, Hunter New England Local Health District, Longworth Ave, Wallsend, NSW Australia; 5 The George Institute for Global Health, Level 3, 50 Bridge St, Sydney, NSW 2000 Australia; 6 Dalla Lana School of Public Health, University of Toronto, 155 College St, Toronto, ON M5T 3M7 Canada; 7 Institute of Regional Health Services Research, University of Southern Denmark, Winsloewparken 193, DK-5000 Odense C, Denmark; 8 Sports Medicine Clinic, Orthopaedic Department Hospital of Lillebaelt, Østre Hougvej 55, DK-5500 Middelfart, Denmark

**Keywords:** Spinal pain, Children, Adolescents, Prevalence

## Abstract

**Background:**

Spinal pain in children and adolescents is a common condition, usually transitory, but the picture of spinal pain still needs elucidation, mainly due to variation in measurement methods. The aim of this study was to describe the occurrence of spinal pain in 8–15 year-old Danish school children, over a 3-year period. Specifically determining the characteristics of spinal pain in terms of frequency and duration.

**Methods:**

The study was a 3-year prospective longitudinal cohort study including 1400 school children. The outcomes were based on weekly text messages (SMS) to the parents inquiring about the child’s musculoskeletal pain, and on clinical data from examinations of the children.

**Results:**

The 3-year prevalence was 55%. The prevalence was 29%, 33% and 31% for each of the three study years respectively, and increased statistically significantly with age, especially for lumbopelvic pain. Most children had few and short-lasting episodes with spinal pain, but more than one out of five children had three or more episodes during a study year and 17% of all episodes lasted for more than 4 weeks.

**Conclusion:**

This study demonstrates that spinal pain is a substantial problem. Most episodes are brief, but there are a vast number of children with frequent and long-lasting episodes of spinal pain indicating a need for action regarding evidence-based prevention and management.

**Electronic supplementary material:**

The online version of this article (doi:10.1186/s12891-017-1424-5) contains supplementary material, which is available to authorized users.

## Background

There is growing evidence that spinal pain in children and adolescents is a common condition, usually transient, self-limiting and rarely associated with serious identifiable pathology [1, 2]. However, we know that children with spinal pain are more likely to become adults with spinal pain [3, 4], and the lifetime prevalence increases steadily to reach adult levels around the age of 18 [3, 5]. This is a challenge to both individuals and societies because of the associated personal and economic burdens.

Unfortunately, it is difficult to obtain a comprehensive picture of the extent of spinal pain due to variation in the manner in which adolescent spinal pain is reported across different studies. Sources of variability between studies include bodily area, duration of episode and definition of recurrences [2, 5, 6]. There is also variation in measurement methods, particularly relating to length of recall, and whether or not a pain severity threshold is set [5, 7]. These reasons likely explain why prevalences reported in studies vary widely, ranging from 1 to 89% [1, 2, 7, 8].

The course of spinal pain in childhood and adolescence is also still unclear, but there seems to be a certain age at which the onset of spinal pain is most common [6, 9], and we also know that the prevalence of spinal pain increases with age [6, 10]. In addition, knowledge about consequences of spinal pain is limited [11], as is knowledge about duration and frequency of pain episodes. Of particular interest is a smaller group of individuals who appear to have recurrent and more painful spinal pain events [2, 3], especially considering that the teenagers with the most frequent back pain seemed to have the highest risk of back pain in adulthood [2–4, 12].

Reliable understanding of prevalence and course of spinal pain is essential for further research into the development of effective prevention and treatment strategies [11]. This study will extend our understanding in the area by capturing accurate estimates of prevalence, number of episodes and length of episodes with spinal pain in children and adolescents aged 8 to 15 years.

The overall aim of this study was to describe the characteristics of spinal pain episodes in 8–15 year-old Danish school children followed for three study years. Specifically we aimed to:

1. Calculate the proportion of individuals reporting any type of spinal pain during a study year
2. Report the prevalence, frequency and duration of spinal pain by means of:
  - The proportion of weeks with spinal pain per study year per child
  - The number of spinal pain episodes per study year per child
  - The length of spinal pain episodes per study year
  - The relationship between number of episodes and episode length per study year per child
3. Determine the relationship between episode length and pain site (cervical, thoracic or lumbopelvic pain), and episode length and complaint severity

## Method

### Overview of design

This study was a 3-year prospective longitudinal cohort study of school children who took part in the Childhood Health, Activity and Motor Performance School Study (CHAMPS Study-DK). The protocol for CHAMPS Study-DK has been published elsewhere (14). The main purpose of the CHAMPS Study-DK was to evaluate the influence of extra physical education (PE) on general childhood health including musculoskeletal complaints. The schools were divided into two groups: one receiving the normal amount of two PE lessons per week (control) and the other receiving six PE lessons per week (intervention). The study involved researchers with a range of professional backgrounds, all investigating different aspects of childhood health.

The CHAMPS Study-DK commenced in 2008 and the data collection regarding injuries and back problems ended in summer 2014. The study was an open cohort study and children could enter or leave the study at any time during the study period. Originally, the study was designed to last for 3 years (2008–2011), but additional funding made it possible to continue for 3 more years. Another team of researchers were responsible for the additional 3 years, which constitutes the basis for this study.

### Participants and setting

Participants in this study included children aged 8–15 from 13 primary schools in the municipality of Svendborg, Denmark. Svendborg consists of approximately 58,000 inhabitants and is considered representative of the Danish population [13]. The schools were matched according to the size and distribution of the socio-economic groups within the uptake area. The clinical team responsible for the follow-up consisted of experienced chiropractors, physiotherapists and a medical doctor.

At baseline, the children and their parents filled out a questionnaire with information on age, sex, health status, parental educational level, work and leisure time activities.

### Outcome measurement

Outcomes were captured via weekly text messages (SMS) to one of the parents of participating children, inquiring about the child’s musculoskeletal complaints, and the amount and type of leisure time sports activity during the past week (see Additional file 1). It was only possible to connect one telephone number to the SMS system and as the phone number was a personal mobile it was generally the same parent answering throughout the study period. Answers were automatically registered, entered and stored in a database. If the parent did not reply, the parent automatically received up to two SMS reminders within the week. The SMS-response is a very efficient way to obtain information on a frequent basis [14, 15]. There were no text messages during the summer and Christmas holidays to reduce the parent’s burden and because there was no possibility of following-up on positive reports of pain.

To avoid break in data continuity due to the long summer break, we chose to report by study year rather than for three full calendar years, i.e. year 1 representing the school year starting in August 2011 and ending in June 2012, year 2 representing the school year starting in August 2012 and ending in June 2013 and year 3 representing the school year starting in August 2013 and ending in June 2014.

In the first SMS question, parents were asked if their child had had any musculoskeletal pain in the previous week. Response options were: ‵1′ for spinal pain, ‵2′ for upper extremity pain, ‵3′ for lower extremity pain, any combination of the three numbers or ‵4′ if there was no pain.

If musculoskeletal pain was reported (response options 1, 2, 3 or any combination of the three numbers), the parents were interviewed by telephone by a member of the screening team. This team was composed of experienced chiropractors and physiotherapists. They administered a standardized interview that included information about the duration of the complaint, the mode of onset, the nature of the pain and any interventions that have been tried (e.g. treatments, drugs used). Based on this interview, complaint severity was classified as trivial or non-trivial.

If the complaint was considered to be non-trivial, an appointment for an examination was made. The examination of non-trivial complaints took place at the child’s school within 2 weeks of first reporting. A member of the clinical team consisting of chiropractors and physiotherapists with extensive experience in examining children performed the examination. Following the examination, complaints were categorized according to the International Classification of Diseases (ICD-10). The child was offered advice on how to handle his/her problem and the parents were notified about the result and any potential action following the examination either by telephone or letter. All data were filed in an electronical journal system established specifically for this project and stored on a secure server.

### Data analysis

STATA 14.0 (StataCorp, College Station, Texas, USA) was used for data analyses. Data for these analyses were collected over 44 weeks in study year 1, 47 weeks in study year 2, and 46 weeks in study year 3, giving a total of 137 weeks.

To obtain a satisfactory observation period, we excluded the children for whom the observation period was less than a study year minus 1 week (from the first SMS to the last SMS), e.g. less than 43 possible answer weeks in study year 1. Within this period there was the possibility of missing answers, and thus we also excluded cases with less than 50% answers within that period to ensure reliable estimates.

1. A 3-year prevalence with 95% confidence intervals (CI) was calculated for the children that participated for the entire study period, including sex-specific prevalences. We calculated the study year specific prevalences for each study year, including sex-specific prevalences. Finally, we calculated the age-specific prevalences for each age from 8 to 15 years old. The relationship between age and prevalence of spinal pain was assessed using test for trend as described by Cuzick [16].
2. The characteristics of spinal pain were described as a) the proportion of weeks with spinal pain, b) the number of episodes, c) the duration of episodes per child and d) relationship between number of episodes and episode length.
  - Proportion of weeks with spinal pain
    The proportion of weeks a child experienced spinal pain was calculated by dividing all answers that included a ‵1′ by the total weeks of observation within a study year. This is illustrated graphically with histograms including medians with interquartile ranges, and means with standard deviations.
  - Number of episodes per child
    A new episode was defined as an episode occurring after at least 1 week without spinal pain. It was reported using numbers and percentages, described with medians with interquartile ranges and means with standard deviations.
    A sensitivity analysis was conducted to assess the effect of the recovery definition, i.e. recovery was defined as 4 weeks of ‘ no pain’ [17, 18], instead of 1 week, before a subsequent episode was considered to be a new episode.
  - Duration of episodes
    The length of an episode was calculated as the number of weeks of continuous reporting ‵1′ (i.e. spinal pain). Because a small number of the children had very long episodes, we chose to truncate episode length at 13 weeks, as this is a commonly used definition of chronic pain [19], and to prevent these few individuals from skewing the results disproportionately. We reported numbers and percentages, medians with interquartile ranges and means with standard deviations. A sensitivity analysis was conducted to assess the effect of the recovery definition, i.e. recovery was defined as 4 weeks of ‘no pain’, instead of 1 week, before a subsequent episode was considered to be a new episode.
  - Relationship between number of episodes and episode length
    The relationship between number of episodes and episode length was assessed using test for trend.
3. Region specific spinal pain diagnoses were made by the clinicians in the subset of children with non-trivial spinal pain. These were coded into painsites, i.e. cervical, thoracic, lumbopelvic or multisite pain (defined as pain in more than one spinal region). If one continuous episode consisted of pain from different spinal regions at different timepoints, the whole episode was considered as multisite.
  Prevalences with 95% CI and episode length (medians with interquartile ranges and means with standard deviations) were reported for the different painsites as well as for trivial vs. non-trivial complaints. Any differences between groups in relation to episode length were evaluated using One-way analysis of variance for complaint type and *t*-test for pain site. Significance level was set to 5%.

### Missing data

Missing SMS responses had an impact on how to determine the length of an episode because it was impossible to determine if the child still had spinal pain or was pain-free in the week with the missing answer. We therefore formulated two decision rules for defining the end of an episode. The first was if there were four or fewer consecutive missing answers, preceded and followed by a ‵1′, then this was considered as one continuous episode and the missing values were imputed as ‵1′. The second was if there were more than four consecutive missing answers, or the next answer after missing was ‵2′, 3′ or ‵4′, we considered the episode of spinal pain as terminated by the last report of ‵1′.

Because there is no literature to support this decision, a sensitivity analyses was performed to estimate the impact of this decision. For that purpose, the missing weeks were treated in two extreme ways: first, we imputed the missing answers to be the same as the last answer, regardless of the value of the next report. This would potentially inflate the episode lengths and diminish the number of episodes. Second, we imputed an answer of ‵4′ (no pain) for all the weeks with missing answers, which would do the opposite. Thereby, we determined the range within which the correct answer would likely lie.

## Results

**Fig. 1:**
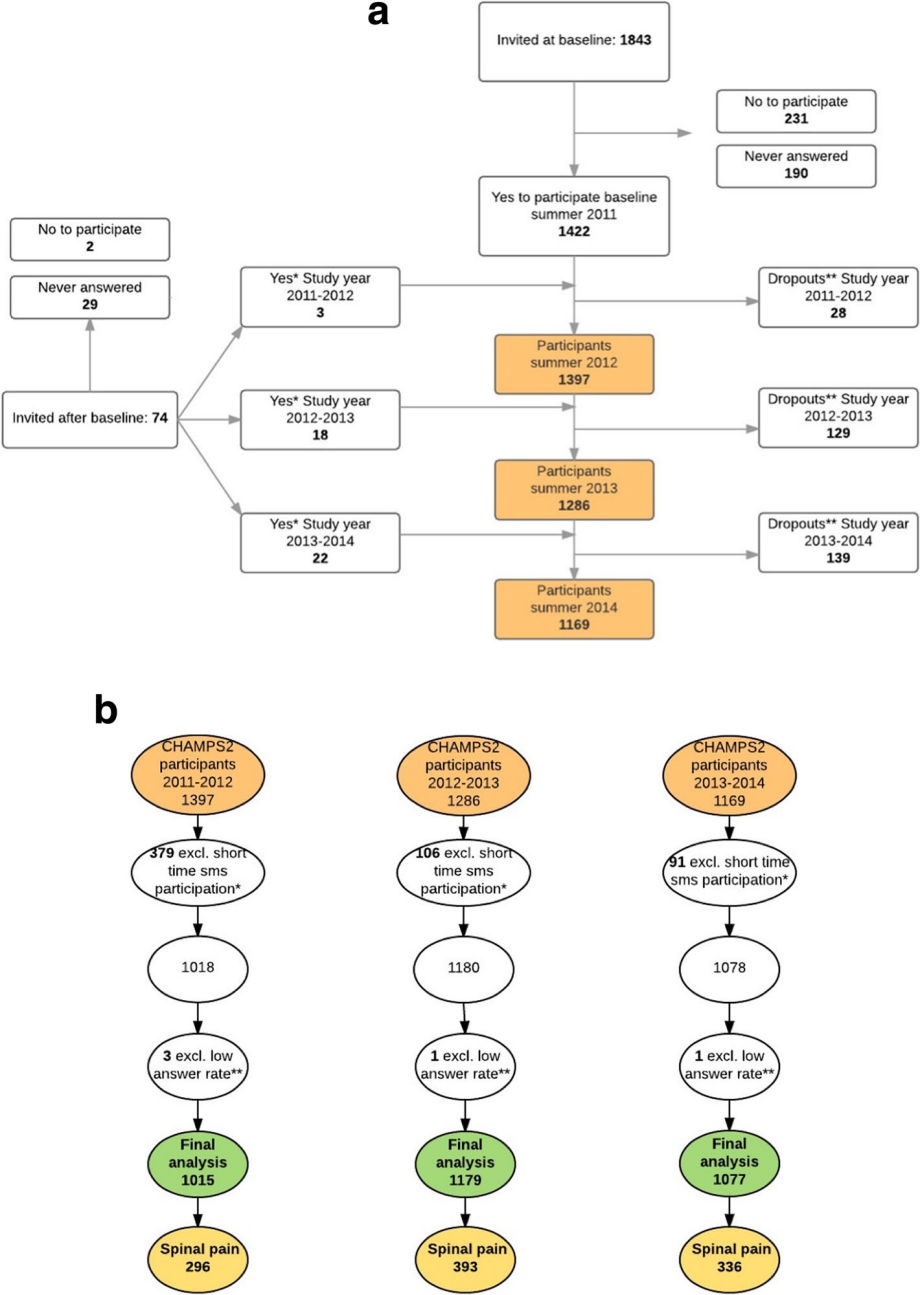
**a** Participant flow CHAMPS 2 2011–2014. *Dropins: change of school or wish to enter the project. **Dropouts: change of school or personal reasons. **b** Participant flow SMS track. *Children participating less than maximum possible number of weeks minus one. **Children answering less than 50% of participation time

In total, 1917 children were invited to participate in the study and 421 either refused to participate or never anwered. Thus, the cohort included 1465 children (766 girls, (52%)) who were followed for up to 3 years, ranging from 1 to 137 weeks (median 137, IQR 110–137). There was a statistically significant difference among schools according to the 3-year prevalence (*p* < 0.001). However, this difference was only driven by study year 2 (*p* = 0.01). There were no differences in study year 1 (*p* = 0.35) and study year 3 (*p* = 0.19). The difference found in study year 2 was based on a high prevalence from two schools, but the same schools did not have high prevalences in the other two study years, and therefore, we consider this to be a chance finding. There was a statistically significant difference (*p* < 0.05) between participants and non-participants in the study according to which school they were attending, but not according to sex. The average weekly SMS response rate for all schools for all 3 years was 96.4% (ranging from 93.7 to 98.3%) with a total of 158,478 observations. Dropouts occurred when children moved away from their school or for personal reasons (Fig. 1a).

Twenty Seven percent of the participants were excluded in study year 1, 8% in study year 2, and 8% in study year 3 because the SMS participation period was too short, and five children were excluded due to low response rate (<50%) (Fig 1b). There were a higher number of children excluded in year 1 because of an administrative change of the school districts. This resulted in new schools being enrolled in the project, and during the first half year the parents gradually consented to let their children participate in the study. Furthermore, the older children from some schools were joined in a special school class on a school that was not part of the project.

After exclusion of those participants, the cohorts used for analyses consisted of 1015 participants in study year 1, 1179 in study year 2, and 1,077 in study year 3 (Table 1). In total, 1327 children (690 girls, 52%,) over the 3 years (2011–2014) were in the cohort and of these, 794 children (416 girls, (52%)) participated for all years.

**Table 1:** Age, sex and type of school for the children participating by study year

	Study year 1 (2011–2012) (*N* = 1015)		Study year 2 (2012–2013) (*N* = 1179)		Study year 3 (2013–2014) (*N* = 1077)	
	Number of children	% girls	Number of children	% girls	Number of children	% girls
Age 8	50	54%	−	−	−	
Age 9	197	61%	69	55%	−	
Age 10	213	50%	236	58%	68	57%
Age 11	224	50%	271	51%	233	59%
Age 12	225	52%	270	47%	244	52%
Age 13	103	49%	218	57%	226	45%
Age 14	3	33%	112	47%	199	53%
Age 15	−	−	3	33%	106	48%
Age 16	−	−	−	−	1	0%
I-school/c-school^a^	602/413	56%/47%	800/518	56%/47%	767/440	55%/48%

^a^I-school: intervention school, 6 h PE per week

C-school: control school, 2 h PE per week

### Prevalence

The 3-year prevalence for spinal pain was 55.5% [95% CI: 52.1–59.0%] for the children who participated in all three study years. No statistically significant difference was found for spinal pain according to sex (girls 58.2% [95% CI: 53.4–62.8%] vs boys 52.6% [95% CI: 47.6–57.6%], *p* = 0.12). There was no statistically significant difference in the prevalence of spinal pain between the children having more PE lessons compared to those with a standard amount of PE lessons. We therefore chose to report on the children as one cohort throughout this study and not take the number of PE lessons into account.

In study year 1, the prevalence for spinal pain was 29.2% [95% CI: 26.4–32.0%], in study year 2 it was 33.3% [95% CI: 30.7–36.1%], and in study year 3 the prevalence was 31.2% [95% CI: 28.5–34.0%]. Girls more often reported neck- and back pain than boys in all 3 years, but the difference was only statistically significant in study year 1 (*p* = 0.01).

**Fig. 2:**
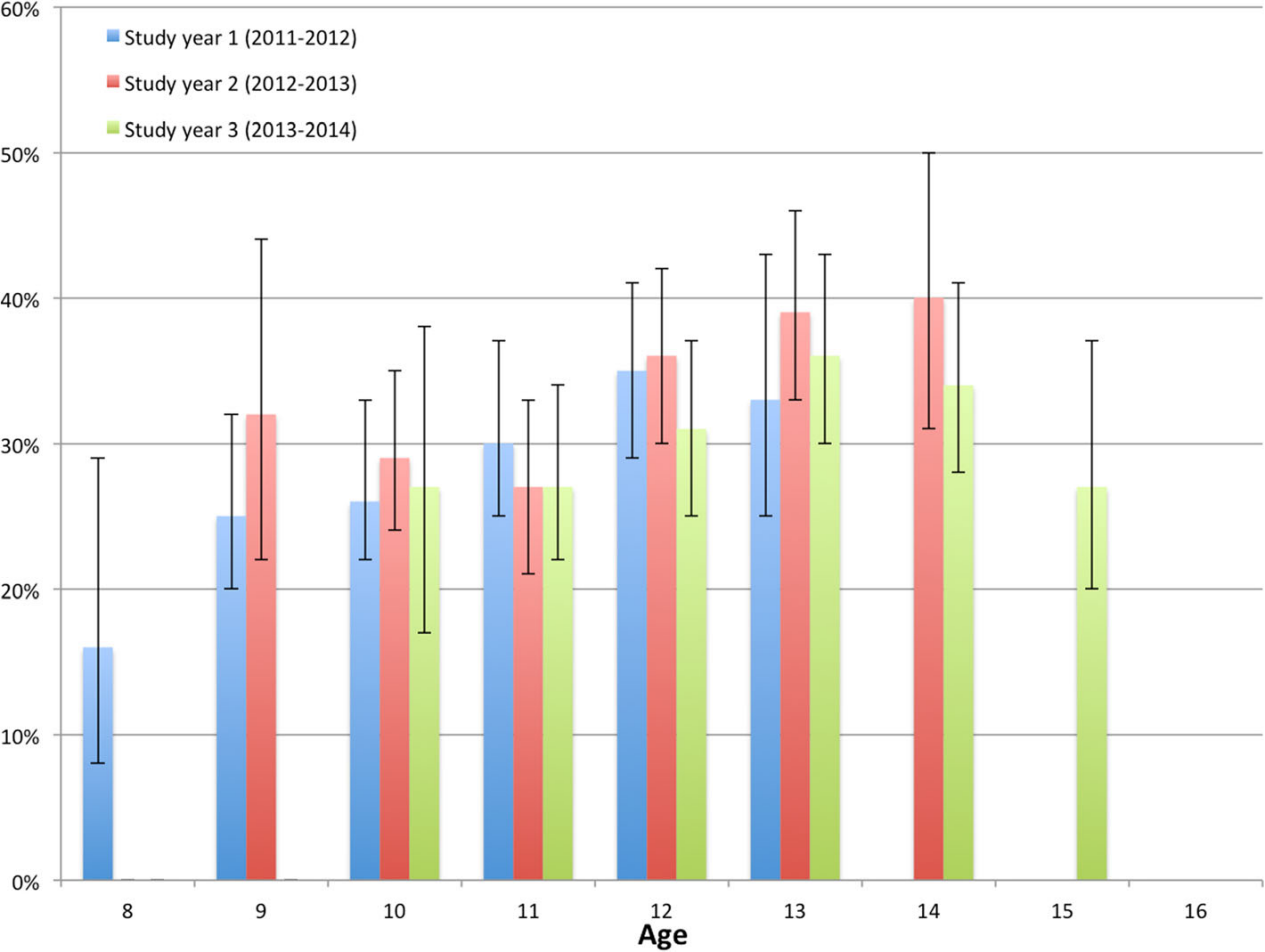
Prevalence by age and study year

Prevalence of spinal pain by age and study year can be seen in Fig. 2, ranging from 16.0% at age eight in study year 1 to 40.2% at age 14 in study year 2. The prevalences generally increased with age, and this was confirmed in the trend test looking at all three study years (*p* < 0.05). The largest increase appeared at age 12 (Fig. 2).

### Proportion of painweeks, number of episodes and lengths of episodes

**Fig. 3:**
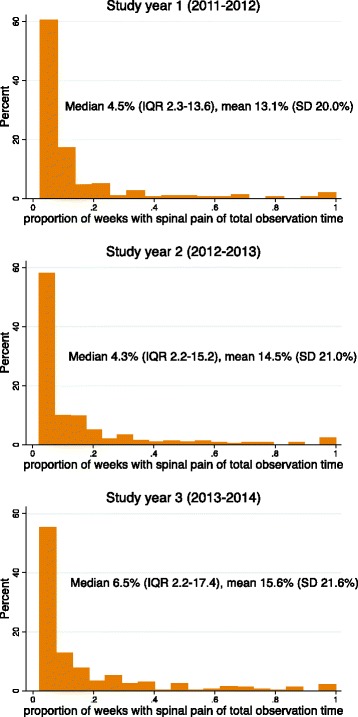
Proportion of weeks with spinal pain by study year. (Proportion of painweeks are not truncated at 13 weeks)

Most children had few weeks with spinal pain during the 3-year study (Fig. 3). Forty-seven to 54% of the affected children had pain for less than 5% of the weeks reported. A small proportion of children had pain for more than 50% of the time (7%, 7% and 8% for study years 1, 2 and 3, respectively).

The majority of the children had one episode by study year (Table 2), but up to one fourth of the children had three or more episodes during a study year (21%, 20% and 25%, respectively for the three study years). In addition, there seemed to be a slight increase in the number of episodes over the 3-year study period.

**Table 2:** Number of episodes per child by study year

Number of episodes	Study year 1 (2011–2012) (*N* ^a^ = 296)		Study year 2 (2012–2013) (*N* ^a^ = 393)		Study year 3 (2013–2014) (*N* ^a^ = 336)	
1	60.1%	178	59.5%	234	55.7%	187
2	18.6%	55	20.6%	81	19.3%	65
3	8.8%	26	7.9%	31	11.3%	38
4	5.4%	16	4.8%	19	6.2%	21
5	4.0%	12	3.3%	13	3.9%	13
6	1.7%	5	1.5%	6	2.4%	8
≥7	1.4%	4	2.4%	9	1.2%	4
	100%	296	100%	393	100%	336
Median # episodes (IQR)	1 (1–2)		1 (1–2)		1 (1–2.5)	
Mean # episodes (SD	1.9 (1.4)		1.9 (1.6)		1.9 (1.4)	

^a^ = Number of children per school year

Most of the episodes were short with 51–59% lasting for 1 week, but 16–17% of the episodes lasted for 5 or more weeks by study year (Table 3). Furthermore, for a significant number of children (10%, 13% and 10%, respectively for the three study years) all episodes were long lasting (5 or more weeks).

**Table 3:** Length of episodes

Length of episode (weeks)	Study year 1 (2011–2012) (*n* ^a^ = 550)		Study year 2 (2012–2013) (*n* ^a^ = 746)		Study year 3 (2013–2014) (*n* ^a^ = 660)	
1	59.1%	325	56.6%	422	51.2%	338
2	13.1%	72	14.7%	110	17.9%	118
3	7.4%	41	6.7%	50	7.3%	48
4	4.4%	24	4.9%	37	6.2%	41
5	3.6%	20	3.1%	23	2.9%	19
6	2.2%	12	1.5%	11	3.5%	23
7	2.2%	12	2.1%	16	1.5%	10
8	1.1%	6	1.5%	11	0.3%	2
9	1.1%	6	0.7%	5	0.4%	3
10	1.1%	6	0.9%	7	1.2%	8
11	1.1%	6	0.3%	2	0.5%	3
12	0.7%	4	0.4%	3	0.6%	4
≥13	2.9%	16	6.6%	49	6.5%	43
	100%	550	100%	746	100%	660
Median # weeks (IQR)	1 (1–3)		1 (1–3)		1 (1–3)	
Mean # weeks (SD)	2.6 (2.9)		2.9 (3.3)		3 (3.3)	

^a^ = Number of episodes per school year

The relationship between number of episodes and mean episode length showed that for the children with only one episode in a study year, 57%, 58% and 64% (respectively for the three study years) of these episodes lasted only 1 week. However, for the children with three or more episodes in a study year, only 38%, 51% and 39% of these episodes lasted for 1 week or less. The test for trend by study year showed a statistical significant difference (*p* < 0001) indicating that the more episodes a child had, the longer the episodes were.

### Regional spinal pain and episodes

In total, 185 different ICD-10 diagnoses were given for the non-trivial spinal pain episodes (e.g. cervicalgia, lumbar facet syndrome, unspecific back pain) and these were classified into mutually exclusive pain sites: 42% lumbopelvic, 31% cervical, 14% thoracic and 13% multisite. Because the data were not normally distributed, a log transformation was performed before the analyses. There was a decreasing number of cervical pain episodes (27.7–22.4%) and an increasing number of lumbopelvic pain episodes (38.5–48.9%) over the 3-year period, but this was not statistically significant, whereas the number of thoracic and multisite pain episodes varied non-systematically (Table 4). The length of episodes did not vary much according to type of regional pain (Table 4), although there was a tendency for multisite pain to last longer (median 3.7, IQR 1–13) and thoracic pain episodes to be shorter (median 2.5, IQR 1–5). The results were only statistically significant for study year 3 (*p* = 0.05).

**Table 4:** Episode lengths in relation to pain site

	Study year 1 (2011–2012) (*N* ^a^ = 148)				Study year 2 (2012–2013) (*N* ^a^ = 248)				Study year 3 (2013–2014) (*N* ^a^ = 223)			
	*n*	% [CI]	Episode length (median, IQR)	Episode length (mean, SD)	*n*	% [CI]	Episode length (median, IQR)	Episode length (mean, SD)	*n*	% [CI]	Episode length (median, IQR)	Episode length (mean, SD)
Cervical pain	41	27.7% [21.0–35.5%]	3 (2–7)	4.9 (3.9)	63	25.4% [20.3–31.2%]	3 (1–6)	4.3 (3.6)	50	22.4% [17.4–28.4%]	2 (1–7)	4.7 (4.7)
Thoracic pain	25	16.9% [11.6–23.9%]	3 (1–5)	4 (3.4)	49	19.8% [15.2–25.2%]	2 (1–4)	3.7 (3.6)	32	14.4% [10.3–19.6%]	2.5 (1–4)	3.5 (3.0)
Lumbopelvic pain	57	38.5% [30.9–46.7%]	3 (2–7)	4.7 (4.5)	107	43.1% [37.1–49.4]	2 (1–6)	4.1 (3.9)	109	48.9% [42.3–55.5%]	3 (2–6)	4.2 (3.8)
Multisite pain	25	16.9% [11.6–23.9%]	3 (1–6)	4.6 (4.5)	29	11.7% [8.2–16.4%]	4 (1–13)	6.7 (5.3)	32	14.3% [10.3–19.6%]	4 (2–13)	6.5 (5)

^a^Number of diagnosed spinal pain episodes per study year

### Trivial vs. non-trivial complaints and episodes

The majority of complaints (approximately 2/3) were of a trivial character, i.e. without a diagnosis, in all three study years (Table 5), but the tendency shifted towards more non-trivial complaints in study year three. Because the data were not normally distributed, a log transformation was performed before the analyses. The episodes were statistically significantly longer for the non-trivial complaints when compared to the trivial complaints in all three study years (*p* < 0.001), but medians and means did not change according to study year (Table 5).

**Table 5:** Complaint type according to episode length

	Study year 1 (2011–2012) (*N* ^a^ = 550)				Study year 2 (2012–2013) (*N* ^a^ = 746)				Study year 3 (2013–2014) (*N* ^a^ = 660)			
	*n*	% [CI]	Episode length (median, IQR)	Episode length (mean, SD)	*n*	% [CI]	Episode length (median, IQR)	Episode length (mean, SD)	*n*	% [CI]	Episode length (median, IQR)	Episode length (mean, SD)
Trivial pain	405	73.1% [69.2–76.6%]	1 (1–2)	1.8 (2.0)	498	66.8% [63.2–70.1%]	1 (1–2)	2.1 (2.6)	437	66.2% [62.5–69.7%]	1 (1–2)	2.1 (2.4)
Non-trivial pain	148	26.9% [23.3–30.8%]	3 (1–7)	4.7 (3.9)	248	33.2% [29.9–36.7%]	3 (1–6)	4.4 (4.0)	223	33.8% [30.3–37.5%]	3 (2–6)	4.5 (4.2)

^a^ = Total number of spinal complaints pr year

### Sensitivity analyses

Results of the sensitivity analysis assessing the impact of missing data showed no differences between the three different types of imputation in relation to number and lengths of episodes (Table 6).

**Table 6:** Sensitivity analyses on missing data

	Primary data				v1 data				v2 data			
	Median number of episodes (IQR)	Mean number of episodes (SD)	Median length of episodes (IQR)	Mean length of episodes (SD)	Median number of episodes (IQR)	Mean number of episodes (SD)	Median length of episodes (IQR)	Mean length of episodes (SD)	Median number of episodes (IQR)	Mean number of episodes (SD)	Median length of episodes (IQR)	Mean length of episodes (SD)
Study year 1	1 (1–2)	1.9 (1.4)	1 (1–3)	2.6 (2.9)	1 (1–2)	1.9 (1.4)	1 (1–3)	2.6 (2.9)	1 (1–2)	2.0 (1.5)	1 (1–3)	2.7 (3.0)
Study year 2	1 (1–2)	1.9 (1.6)	1 (1–3)	2.9 (3.3)	1 (1–2)	1.9 (1.6)	1 (1–3)	2.9 (3.3)	1 (1–2)	2.0 (1.7)	1 (1–3)	2.9 (3.3)
Study year 3	1 (1–2.5)	2.0 (1.4)	1 (1–3)	3.0 (3.3)	1 (1–3)	2.0 (1.4)	2 (1–3)	3.0 (3.3)	1 (1–3)	2.1 (1.5)	2 (1–4)	3.0 (3.3)

- Primary data: up til 4 missing weeks after a‵1′ is imputed with‵1′
- v1: all missing weeks after a‵1′ is imputed with‵1′
- v2: all missing weeks after a‵1′ is imputed with‵4′

Defining a new episode as starting after 4 weeks of ‘no pain’ instead of 1 week, resulted in a reduction of number of episodes by 20.0%, 18.8% and 18.0% in study years 1, 2 and 3 respectively, and the maximum number of episodes decreased from 8 to 5, 12 to 6 and 9 to 6 in study years 1, 2 and 3 respectively. No difference in the median number of episodes was found and the mean number was only slightly smaller (1.9 to 1.5), with a higher proportion of children having 1 or 2 episodes.

Finally, we found somewhat higher proportion of episodes lasting for 1 week, (62.0%, 59.1% and 53.2% vs 59.1%, 56.6% and 51.2% for study year 1, 2 and 3 respectively), but overall, the distribution between the different lengths of episodes was almost the same.

## Discussion

This study reports weekly spinal pain in children and adolescents with up to 3 years of follow-up in a large cohort. Spinal pain was experienced by approximately half of the children at some point throughout the 3-year study period and the 1-year prevalence approximated 30%. Most children had few and short episodes of pain, but a rather substantial number of children had more frequent and longer lasting episodes. The prevalence of spinal pain increased significantly with age. There was no statistically significant difference in spinal pain prevalence between children having two or six PE lessons. This was indeed an interesting finding, but not the aim of this study and therefore we did not analyse this further, but will probably include it in a future manuscript.

This study reported a slightly higher 1-year prevalence than a study using the same cohort (30% vs. 25%) 3 years earlier [1]. This confirms the finding of increasing prevalence with age as found in the current study. Likewise, it is consistent with the observations in a meta-analysis by Calvo-Munoz [7] (mean overall prevalence 33%), who also reported an increase in prevalence with increasing age despite considerably different methodologies in studies and potential recall bias from studies commonly reporting 1-year prevalence recalls. The increase from around age 12, which has also been shown in other studies [9, 20], indicates that this could be an important age regarding prevention and/or treatment.

We do not know much about the impact of adolescent spinal pain on general health, but Gobina et al. showed a strong association between the use of pain medication and recurrent low back pain in adolescents [8]. In addition, Hestbaek et al. reported that adolescents with low back pain have more comorbidity than adolescents without low back pain [21]. We are unable to determine if these issues are present in our cohort or the impact that spinal pain may have on our adolescents’ general health. However, these issues should give rise to extra concern about recurrent spinal pain in this age group. Considering the association between low back pain in adolescence and low back pain in adulthood, recurrent spinal pain in this age group also presents a potentially significant health challenge in their adult years [3].

Similar to other studies, we found that most children had a few short episodes of pain [1, 2]; however, a significant number of children did have pain more often and for longer periods of time. Of those with spinal pain, 20–25% in our study had three or more episodes during a study year and 16–17% of all episodes lasted for more than 4 weeks, indicating that recurrent or persistent spinal pain is not uncommon in this age group. This is similar to previous studies that reported rates of persistent low back pain in adolescents (14–26%) [2, 8, 12, 22, 23].

Defining episode length based on 1 or 4 weeks of ‘no pain’ between episodes resulted in only minor differences in the median and mean episode lengths and thus did not introduce a systematic bias to the results. These findings are in line with other studies suggesting, that 1-month without back pain would be an appropriate cutpoint [17, 18].

Due to the subjective judgement of the telephone interviewers, there is a potential risk for misclassification of the complaints. Fortunately this only relates to a small part of the study (last part of objective 3) and therefore does not affect our primary objective of prevalence.

Another potential source or error could have been the parents’ response to the SMS question. In order to avoid a phone call from a clinician following a pain report, parents may have reported ‘no pain’ despite actual pain reports from the child, which would have caused an under-reporting of spinal pain. Furthermore, it could be a concern that the children and their parents might have changed their behaviour of reporting pain during the study period, since they have answered SMS-questions continously for up to 6 years. However, when comparing to another Danish project with school children (aged 11–15) who were not followed with SMS, but simply answered one questionnaire, the prevalences (lifetime prevalence 86%, 1-week prevalence 36% and point prevalence 17%) seem to be comparable with our results [2]. In addition, the proportion of missing weeks did not increase by study year, indicating continued dedication to the project.

Finally, nested in this cohort was a randomised clinical trial, which compared two different kinds of manual treatment, and all of the children enrolled in the trial received more clinical care than usual [24]. We do not know how this might have impacted the overall prevalence and characteristics of the spinal pain episodes. We have little knowledge (sex and school only) about the children that refused to participate in the study. We did find that the refusal rate differed across schools, and therefore bias is likely to be non-differential in relation to back pain, but the generalizability might be compromised.

The parents’ answer may not have been a good proxy for the child’s true health status, especially in the context of the development from child to adolescent. Kamper et al.[25] did a study on the same cohort investigating the agreement between the child’s own assessment of their pain and the parents’ report of their child’s pain, and found that the child expressed pain more often than the parents. However, when the parents did report pain, the child also reported pain, which indicated that the parents did not over-report pain. The same pattern was found by Sundblad et al.[26]. For our study, these findings imply that the actual prevalence of spinal pain and the length of spinal pain episodes might have been higher if the children had self-reported, but on the other hand we avoided reports on minor complaints e.g. bruises.

The major strength of this study was the 3-year weekly follow-up in the same cohort using the SMS-track system to collect the outcome measures. The SMS-track system is a very efficient method, providing a very easy way of collecting frequent follow-up. It minimized the recall bias because the parents reported events of the last 7 days; everybody in Denmark has a cell phone; it was easy for everybody to answer; and the response rate was very high. Furthermore, missing responses from the SMS-track system was not an issue. We imputed the missing data using different strategies, and we only found a small difference according to imputation method and study year. These differences did not have an impact on the number and the length of the episodes of spinal pain.

Finally, we combined the SMS track data from the parents with data from the clinicians, which gave us a very complete picture of the frequency, the duration and the localisation of spinal pain.

## Conclusion

Although rates of spinal pain report were high, for most children the pain was short-lived and did not recur frequently. Of concern though, was the rather substantial number of children who reported either persistent or recurrent pain. In at least a quarter of those with spinal pain, the episodes lasted for more than 4 weeks and/or occurred three times or more during a study year. It is towards this group that a concerted research effort is needed to inform evidence-based prevention and management.

## Additional file

Additional file 1: SMS questions. (DOCX 41 kb)

## Abbreviations

CHAMPS: The childhood health, activity and motor performance school study
PE: Physical education
SMS: Short message system (text messages)
